# High Genetic Diversity of *Plasmodium falciparum* in the Low-Transmission Setting of the Kingdom of Eswatini

**DOI:** 10.1093/infdis/jiz305

**Published:** 2019-07-04

**Authors:** Michelle E Roh, Sofonias K Tessema, Maxwell Murphy, Nomcebo Nhlabathi, Nomcebo Mkhonta, Sibonakaliso Vilakati, Nyasatu Ntshalintshali, Manik Saini, Gugu Maphalala, Anna Chen, Jordan Wilheim, Lisa Prach, Roly Gosling, Simon Kunene, Michelle S. Hsiang, Bryan Greenhouse

**Affiliations:** 1 Malaria Elimination Initiative, Institute of Global Health Sciences, University of California, San Francisco; 2 Department of Epidemiology and Biostatistics, University of California, San Francisco; 3 Division of HIV, Infectious Diseases, and Global Medicine, Department of Medicine, University of California, San Francisco; 4 Department of Pediatrics, University of California, San Francisco; 5 National Malaria Programme, Ministry of Health, Manzini; 6 Clinton Health Access Initiative, Mbabane, Eswatini; 7 Ministry of Health, Mbabane, Eswatini; 8 Department of Pediatrics, University of Texas Southwestern Medical Center, Dallas; 9 Chan Zuckerberg Biohub, San Francisco, California

**Keywords:** malaria, malaria elimination, Eswatini, Swaziland, population genetics, microsatellite genotyping, parasite diversity, transmission intensity

## Abstract

**Background:**

To better understand transmission dynamics, we characterized *Plasmodium falciparum* genetic diversity in Eswatini, where transmission is low and sustained by importation.

**Methods:**

Twenty-six *P. falciparum* microsatellites were genotyped in 66% of confirmed cases (2014–2016; N = 582). Population and within-host diversity were used to characterize differences between imported and locally acquired infections. Logistic regression was used to assess the added value of diversity metrics to classify imported and local infections beyond epidemiology data alone.

**Results:**

Parasite population in Eswatini was highly diverse (expected heterozygosity [H_E_] = 0.75) and complex: 67% polyclonal infections, mean multiplicity of infection (MOI) 2.2, and mean within-host infection fixation index (F_WS_) 0.84. Imported cases had comparable diversity to local cases but exhibited higher MOI (2.4 vs 2.0; *P* = .004) and lower mean F_WS_ (0.82 vs 0.85; *P* = .03). Addition of MOI and F_WS_ to multivariate analyses did not increase discrimination between imported and local infections.

**Conclusions:**

In contrast to the common perception that *P. falciparum* diversity declines with decreasing transmission intensity, Eswatini isolates exhibited high parasite diversity consistent with high rates of malaria importation and limited local transmission. Estimates of malaria transmission intensity from genetic data need to consider the effect of importation, especially as countries near elimination.

## Teaser

In contrast to the commonly held perception that *P. falciparum* diversity declines with decreasing transmission intensity, infections from Eswatini exhibited high parasite diversity consistent with high rates of malaria importation and limited local transmission.

The Kingdom of Eswatini, formerly the Kingdom of Swaziland, has made significant progress in reducing its malaria burden and is on track to be one of the first sub-Saharan African countries to eliminate malaria. From 2000 to 2010, Eswatini reduced its malaria cases by 94% [1] and has since sustained very low malaria transmission, reporting an average of 1.5 cases per 1000 between 2010 and 2016 [2]. A key challenge in eliminating malaria in Eswatini has been cross-border migration from neighboring, higher-transmission countries, which has resulted in malaria importation and sporadic onward transmission [3–5]. Between 2014 and 2016, a total of 55% of infections were classified as imported [6], mainly from Mozambique (90%) [4, 7].

To complement traditional approaches to surveillance, genotyping of the *Plasmodium falciparum* parasite has become a popular approach to understanding underlying malaria transmission dynamics [8–10]. The current assumption is that as transmission declines, recombination between genetically distinct clones reduces, leading to decreases in genetic diversity [11]. This relationship, sometimes referred to as a “genomic thermometer” for transmission, has been based on numerous studies that have shown that *P. falciparum* parasite populations generally have higher population and within-host diversity in high-transmission settings of sub-Saharan Africa than in lower-transmission settings (eg, Southeast Asia and Latin America) [8, 11–20] and has been considered a useful method for gauging changes in malaria transmission [8, 21, 22]. However, studies of *P. falciparum* parasite diversity in low-transmission settings of sub-Saharan Africa are limited. In these settings, the relationship between transmission intensity and parasite diversity may demonstrate exceptions to this concept, potentially as a result of importation of malaria from neighboring, higher-transmission countries or recent reductions in transmission.

Here, we evaluate how well the genomic thermometer framework aligns with *P. falciparum* parasite diversity in Eswatini, a low-transmission country challenged by malaria importation from bordering countries with higher malaria transmission, and the utility of parasite diversity metrics to distinguish between imported and locally acquired infections in this setting.

## METHODS

### Study Site

Eswatini is a small, landlocked country in Southern Africa bordered by South Africa and Mozambique, with an estimated population size of 1.3 million [23]. Approximately 30% of the population live in the eastern half of the country, which is still receptive to malaria. In these areas, malaria transmission is unstable and highly dependent on rainfall and cross-border malaria importation [5, 7, 24].

### National Malaria Field Surveillance and Initial Molecular Testing

Malaria is a notifiable disease in Eswatini and all confirmed cases are reported to the national malaria surveillance system [25]. Cases are confirmed by either microscopy or rapid diagnostic test and followed up for investigation, whereby demographics, household GPS coordinates, and travel history are collected. Based on investigation data from the national malaria program, cases are classified as imported if they reported travel to a higher endemic country in the past 8 weeks (excluding travel within the prior week due to a minimum incubation period of 7 days) [26]. If the case resided in a malaria-receptive area, reactive case detection (RACD, ie, focal test and treat using rapid diagnostic tests) is conducted around the household of the index case (ie, the case that triggered the RACD event [27]). Similar epidemiological data are collected from individuals during RACD events and dried blood spots (DBS) are collected from both index and RACD screened cases.

DBS samples underwent molecular testing by loop-mediated isothermal amplification (LAMP) using previously described methods [28]. A second DBS from all index cases and LAMP-positive individuals from RACD was collected for subsequent quantitative polymerase chain reaction (PCR) testing at the University of California, San Francisco (UCSF) using the *var*ATS method [29]. Samples were transported at room temperature and stored at −20°C. In both laboratories, DNA was extracted from DBS using the saponin Chelex method [30].

### Study Population

Study eligibility included cases identified through Eswatini’s national malaria surveillance system between July 2014 and June 2016 (eg, index and RACD cases). Cases had to have a DBS available for genotyping and a parasite density of ≥10 parasites/μL. Ethics approval was granted by the Eswatini Ethics Committee and the UCSF Committee on Human Research.

### Microsatellite Genotyping of P. falciparum

Samples were genotyped using microsatellite markers at 26 loci distributed across the *P. falciparum* genome [11, 31, 32] (Liu et al, in preparation). The neutrality of these 26 loci were evaluated using the F_ST_ outlier detection method under the infinite alleles model [33]. None of the loci showed evidence of strong balancing or positive selection in 602 samples from 26 sub-Saharan African countries (Liu et al, in preparation). To minimize genotyping errors, strict thresholds were used to call alleles using a semisupervised naive Bayes classifier implemented in microSPAT software [34]. Briefly, the software differentiates true peaks from stutter peaks using a classifier algorithm based on the location and size of locus-specific peak patterns relative to a primary peak. Details of the microSPAT software and user setting parameters are provided in Supplementary Table 1. Capillary electrophoresis peaks were excluded if the probability of being a true allele as determined by the algorithm was <95%. Samples genotyped at ≤13 loci were excluded. Data from loci with failed results were treated as missing completely at random and not interpolated with values.

### Characterization of Population-level Diversity

Population-level genetic diversity was assessed by expected heterozygosity (H_E_), number of alleles per locus (ie, allelic richness), and effective population size (N_e_). Values for H_E_ range from 0 to 1 (0 indicating no diversity and 1 indicating all alleles are different). H_E_ was calculated for each locus using the equation $H_{E}=\frac{n}{n-1}[1-\overset{}{\sum }{p_{i}}^{2}]$, where *n* = the number of samples analyzed, $p_{i}$ = the allele frequency of the *i*th allele in the population. Mean H_E_ was calculated by taking the average of H_E_ across all loci. The number of unique alleles per locus (A) is an alternative measure of population-level genetic diversity. The N_e_ was estimated using the infinite alleles model (IAM) and the step-wise mutation model (SMM) as previously described [11]. Genetic differentiation between groups was assessed by comparing H_E_ differences using the Hedrick G′ _ST_ method [35].

To compare Eswatini’s *P. falciparum* genetic diversity relative to other malaria-endemic countries, we conducted a literature search of studies that used a similar subset of microsatellite markers and only studies that published expected heterozygosities at each loci were included. Due to the lack of interlaboratory calibration and nonstandardized allele calling practices of capillary-based microsatellite data, estimates of population-level diversity from Eswatini were recalculated by rescoring alleles based on the exact allele calling methods described in each comparator study, using only the subset of microsatellite markers that were genotyped in both the Eswatini and comparator studies.

### Characterization of Within-Host Diversity

Within-host diversity was assessed by multiplicity of infection (MOI), proportion of polyclonal infections, and within-host infection fixation index (F_WS_). MOI was defined as the second highest number of alleles observed in any of the 26 loci, unless otherwise stated. An infection was considered polyclonal if MOI >1. The F_WS_ metric is a measure of within-host diversity that describes the relationship between the genetic diversity of an individual infection relative to the genetic diversity of the parasite population [36]. A low F_WS_ indicates high within-host diversity relative to the population. F_WS_ was calculated for each sample using the equation $F_{WS}=1-\frac{H_{w}}{H_{s}}$, where $H_{w}\;$ = allele frequency of each unique allele at a particular locus for each individual, and $H_{s}\;$ = heterozygosity of the local parasite population. Amplification bias of different-sized amplicons was corrected by estimating preferential bias as a function of amplicon size difference using known controls at known relative densities [34]. Using previously described F_WS_ thresholds [15, 37], samples with F_WS_ >0.95 were considered clonal infections and samples with F_WS_ ≤0.70 were considered highly diverse infections.

### Predictive Ability of MOI and F_WS_ to Discriminate Between Imported and Local Cases

Logistic regression was used to assess whether the addition of MOI and mean F_WS_ to epidemiologically parameterized models would predict whether a case was imported or locally acquired. The base epidemiological model included the following predictors: district of residence, season, case detection method (passive vs reactive surveillance), age, sex, and occupation. Model improvement was tested using the log-likelihood *Χ*^2^ statistic and discriminative ability was assessed by the change in the area under the receiver operating characteristic curve.

### Sensitivity Analyses

Sensitivity analyses were conducted to assess whether results obtained from genotyped cases were representative of all cases detected during the 2014–2016 period. Due to missing DBS samples or genotype failures, 298 cases were not genotyped, which may have resulted in a nonrandom sampling of the original case population (n = 880). Comparisons between genotyped and nongenotyped cases revealed these 2 groups significantly differed by case detection method (passive vs reactive surveillance), season, district, case classification (imported vs local), sex, and occupation (Supplementary Table 2). To account for this potential source of selection bias, we calculated a propensity score using a logistic regression model [38] with the aforementioned demographic variables to estimate the probability of each case being genotyped. The inverse of these probabilities were used to reweight within-host diversity measures obtained from genotyped individuals to represent the total case population. Other than 3 nongenotyped cases that had a propensity score <0.17, density plots showed good overlap of the propensity scores between genotyped and nongenotyped groups (Supplementary Figure 1), suggesting propensity score reweighting was an appropriate approach to obtain generalizable estimates. None of the results were materially affected by this reweighting, therefore the primary results presented below all use the original, unweighted data.

### Statistical Tests

All analyses were performed using Stata 14.0 (StataCorp, College Station, TX) and R (version 3.5.0; R Project for Statistical Computing; http://www.r-project.org/). Statistical comparisons were conducted using Pearson *Χ*^2^ test for categorical variables, and Student *t* tests or Mann-Whitney tests for continuous variables, depending on the degree of normality of underlying distributions. The Spearman rank correlation (ρ) was used to assess the correlation between mean F_WS_ values and MOI. *P* values < .05 were considered statistically significant.

## RESULTS

### Characteristics of Samples and Genotyping

**Figure 1.**
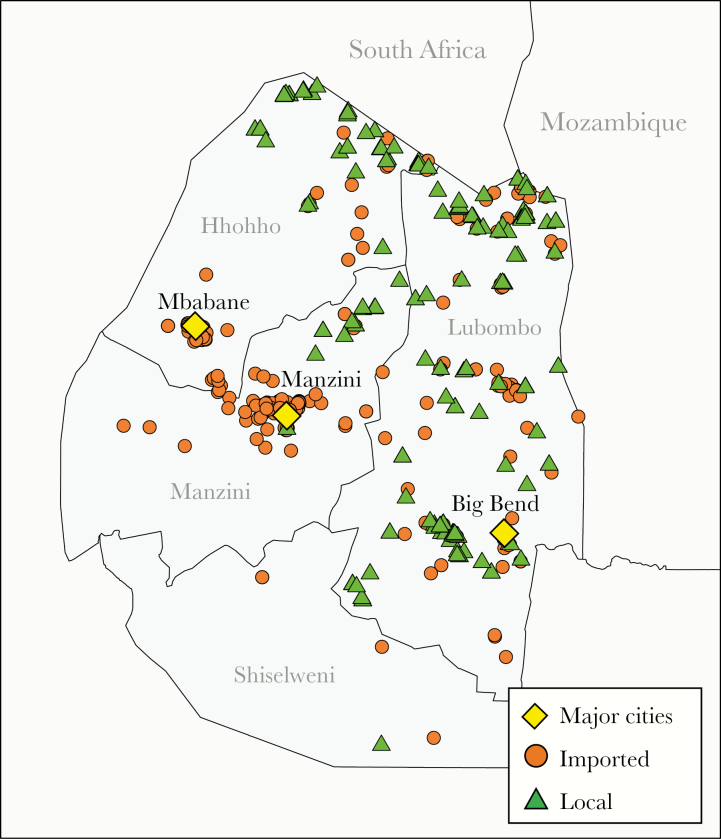
Map of Eswatini cases detected between July 2014 and June 2016. Orange circles represent imported cases and green triangles represent local cases. Yellow diamonds designate major Eswatini cities.

**Figure 2.**
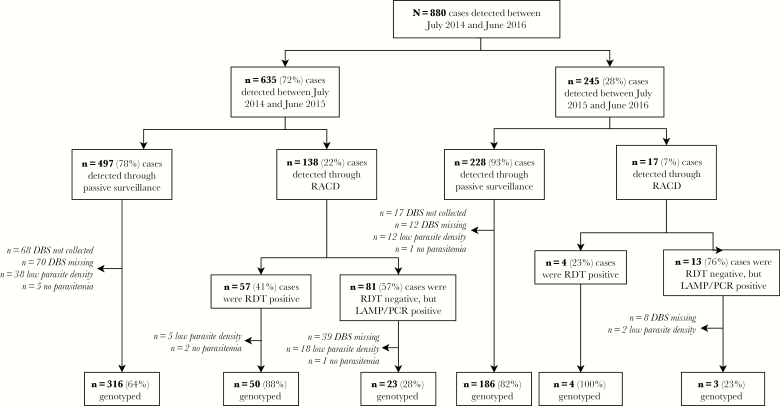
Flow chart of Eswatini samples collected and genotyped between July 2014 and June 2016. Abbreviations: DBS, dried blood spot; LAMP, loop-mediated isothermal amplification; PCR, polymerase chain reaction; RACD, reactive case detection; RDT, rapid diagnostic test.

Between July 2014 and June 2016, 880 *P. falciparum* cases were identified through Eswatini’s national malaria surveillance program (Figure 1 and Figure 2). Between 2014–2015 and 2015–2016, Eswatini experienced a 2.6-fold reduction in cases (from 635 to 245 cases). The majority of cases were detected through passive surveillance (82%) and the rest were identified through RACD. Of the cases identified, 666 *P. falciparum*-positive samples were available for genotyping. The mean success rate across all 26 markers was 86% (range, 74–94). Of the 666 genotyped samples, 582 (87%) samples were genotyped successfully at >13 loci and included in the study. Sixty-six percent of genotyped samples had full coverage of 26 loci. Of the 298/880 cases that were not genotyped, 9 had a parasite density of zero by quantitative PCR (3%) and 75 (25%) had a parasite density below the genotyping threshold (ie, <10 parasites per μL of blood). The remaining 214 samples were not genotyped due to missing DBS samples (Figure 2). In a sensitivity analysis, no differences in population and within-host diversity were observed between all samples included in the study and the subset of samples with full coverage of all 26 loci (data not shown).

The characteristics of the sample population are presented in Supplementary Table 2. Sixty-two percent of genotyped cases (n = 359) were classified as imported by National Malaria Program surveillance officers using self-reported travel history. Of the imported cases that had available country-level travel data (n = 188), 93% reported traveling to Mozambique, with a few cases reporting travel to South Africa (n = 5), Zambia (n = 2), Zimbabwe (n = 2), or Burundi (n = 2). Two-hundred and fifteen infections were classified as local infections and only 12 of these were classified as intraported (ie, imported from different areas within Eswatini [5]).

### Parasite Populations in Eswatini are Complex and Diverse

Polyclonal infections were found in 66% of cases. Mean MOI was 2.2 (range, 1–5) (Table 1). Mean F_WS_ [15, 36], whereby a low F_WS_ demonstrates a higher within-host diversity, in the sample population was 0.84 (range, 0.44–1.00) and was negatively correlated with MOI (Supplementary Figure 2). Thirty-eight percent of cases had a F_WS_ value >0.95 (threshold used to indicate clonal infections) and 23% had a F_WS_ ≤0.70 (threshold for infections exhibiting high within-host diversity) [15, 37]. At the population-level, parasite isolates from Eswatini were highly diverse (mean H_E_, 0.75; range, 0.52–0.93) (Table 2; Supplementary Table 3) and displayed high allelic richness (mean A, 15.4, SD, 7.1; range, 7–32) (Supplementary Table 4). The estimated N_e_ was 4717 (95% confidence interval [CI], 2027–10 745) and 11792 (95% CI, 5067–26 863) under IAM and SMM models, respectively.

**Table 1.** Metrics of Within-Host Diversity in Eswatini by Group

Groups	% Polyclonal (95% CI)	*P* Value^b^	Mean MOI ± SD (Range)	*P* Value^b^	Mean F_WS_ ± SD (Range)	*P* Value^b^
Total (N = 582)	67 (63–70)	…	2.2 ± 1.2 (1.0–7.0)	…	0.84 ± 0.15 (0.44–1.00)	…
Season		.34		.15		.24
June 2014–July 2015 (n = 389)	65 (60–70)		2.2 ± 1.2 (1.0–7.0)		0.84 ± 0.16 (0.44–1.00)	
June 2015–July 2016 (n = 193)	69 (63–76)		2.4 ± 1.2 (1.0–6.0)		0.83 ± 0.15 (0.49–1.00)	
Districts		.032		.30		.11
Hhohho (n = 115)	57 (47–65)		2.1 ± 1.3 (1.0–7.0)		0.87 ± 0.15 (0.49–1.00)	
Lubombo (n = 235)	70 (64–75)		2.1 ± 1.0 (1.0–6.0)		0.83 ± 0.15 (0.47–1.00)	
Manzini (n = 158)	70 (62–76)		2.4 ± 1.3 (1.0–6.0)		0.83 ± 0.16 (0.44–1.00)	
Shiselweni (n = 5)	40 (12–77)		1.8 ± 1.3 (1.0–4.0)		0.86 ± 0.19 (0.58–1.00)	
Passive versus reactive case detection		1.00		.87		.73
Passive (n = 502)	67 (62–71)		2.2 ± 1.2 (1.0–7.0)		0.84 ± 0.15	
Reactive (n = 80)	66 (55–76)		2.2 ± 1.2 (1.0–6.0)		0.83 ± 0.16	
Case classification		.004		.004		.03
Imported (n = 359)	71 (66–76)		2.4 ± 1.3 (1.0–7.0)		0.82 ± 0.16 (0.44–1.00)	
Local^a^ (n = 215)	59 (52–65)		2.0 ± 1.1 (1.0–6.0)		0.85 ± 1.15 (0.47–1.00)	

Abbreviations: CI, confidence interval; F_WS_, within-host diversity relative to that of the population; MOI, multiplicity of infection.

^a^Local infections include indigenous cases (n = 203) and intraported (eg, imported from different areas of the country) cases (n = 12).

^b^All *P* values were 2-tailed and computed using the *Χ*^2^ test for comparing the proportion of polyclonal infections, Poisson regression for MOI, and simple linear regression for F_WS_.

**Table 2.** Metrics of Population-Level Genetic Diversity in Eswatini by Group

Groups	Mean H_E_ ± SD (Range)	Mean A ± SD (Range)
Total (N = 582)	0.75 ± 0.14 (0.52–0.93)	15.4 ± 7.1 (7.0–32.0)
Season		
June 2014–July 2015 (n = 389)	0.75 ± 0.14 (0.54–0.93)	15.0 ± 6.9 (6.0–30.0)
June 2015–July 2016 (n = 193)	0.74 ± 0.16 (0.46–0.94)	12.4 ± 6.6 (4.0–27.0)
Districts		
Hhohho (n = 115)	0.75 ± 0.14 (0.50–0.92)	12.0 ± 5.7 (4.0–22.0)
Lubombo (n = 235)	0.75 ± 0.14 (0.52–0.93)	13.9 ± 6.5 (6.0–29.0)
Manzini (n = 158)	0.75 ± 0.15 (0.50–0.93)	12.7 ± 6.3 (4.0–26.0)
Shiselweni (n = 5)	0.72 ± 0.19 (0.34–0.97)	3.2 ± 1.3 (2.0–6.0)
Passive versus reactive case detection		
Passive (n = 502)	0.75 ± 0.14 (0.52–0.93)	15.3 ± 7.1 (7.0–31.0)
RACD (n = 80)	0.75 ± 0.14 (0.51–0.94)	10.8 ± 6.1 (4.0–27.0)
Case classification		
Imported (n = 359)	0.75 ± 0.15 (0.51–0.94)	14.5 ± 7.0 (5.0–30.0)
Local^a^ (n = 215)	0.75 ± 0.14 (0.49–0.91)	13.2 ± 6.2 (4.0–25.0)

Abbreviations: A, number of unique alleles per locus; H_E_, expected heterozygosity; RACD, reactive case detection.

^a^Local infections include intraported infections (n = 12) and indigenous cases (n = 203).

Despite a 2.6-fold reduction in cases, within-host and population-level diversity were comparable between July–June seasons of 2014–2015 and 2015–2016. Genetic differentiation between seasons was low (G′ _ST_ = 0.026). No significant differences were observed between districts (G′ _ST_ = 0.0068), except in Hhohho and Shiselweni districts, where the proportion of monoclonal infections were higher (50/115 or 43% and 3/5 or 60%, respectively) than in Manzini (71/235; 30%) and Lubombo districts (48/158; 30%) (*P* = .032). Within-host, population-level diversity, and genetic structure did not differ between passively versus reactively detected cases (Table 1; G′ _ST_ = 0.003).

### Genetic Diversity of Parasites in Eswatini is Similar to Higher-Transmission Countries

To assess the relative genetic diversity of Eswatini to that of other malaria-endemic countries, estimates derived from Eswatini cases were compared to other genotyping studies using an overlapping subset of microsatellite markers. Our results demonstrate that mean H_E_ in Eswatini was significantly and consistently higher than those calculated among regions with similarly low malaria transmission intensities (eg, Thailand [18], Vietnam [39], Honduras [40], and Indonesia [41]) and was more comparable to that of higher-transmission countries (eg, Guinea [14], The Gambia [12], and Mali [13]) (Table 3). Comparison of mean MOI across countries showed that estimates from Eswatini were consistently comparable to estimates obtained from higher-transmission areas including in Ghana [43], Mali [15], and Burkina Faso [15] (Table 4). Among the countries listed in Table 4, the proportion of polyclonal infections was positively correlated with malaria incidence, except in Eswatini, where the proportion of polyclonal infections was higher than all other comparator countries despite having very low malaria incidence.

**Table 3.** Comparison of Mean H_E_ Observed in Eswatini Isolates With Previously Published Studies

Country	Year	Annual Confirmed Cases per 1000^a^	H_E_		No. of Loci Compared	*P* Value^c^	Reference, Publication Year
			Country	Eswatini^b^			
Nicaragua	2011	0.1	0.47	0.87	3	.003	Larrañaga et al, 2013 [40]
Thailand	2002–2007	0.5	0.65	0.87	6	.01	Pumpaibool et al, 2009 [18]
Vietnam	1996–1997	0.75	0.76	0.87	6	.028	Ferreira et al, 2002 [39]
Honduras	2011	1	0.31	0.88	3	.017	Larrañaga et al, 2013 [40]
Indonesia (Papua)	2011–2014	1.5	0.69	0.87	8	.02	Pava et al, 2017 [41]
Peru	2003–2007	3	0.53	0.91	3	.078	Branch et al, 2011 [42]
Republic of Guinea	2011	8	0.85	0.87	7	.17	Murray et al, 2016 [14]
The Gambia	2005–2009	39	0.83	0.87	7	.06	Mobegi et al, 2012 [12]
Mali	2012–2015	70	0.84	0.86	5	.42	Nabet et al, 2016 [13]

Abbreviation: H_E_, expected heterozygosity.

^a^Estimates from the World Health Organization Malaria Report. Year 2000 incidence estimates are reported for studies conducted before 2000. Malaria incidence in Eswatini between 2014 and 2016 was estimated at 1 case per 1000 person-years.

^b^Eswatini H_E_ recalculated using only identical loci and the same threshold for allele calling in the comparison study.

^c^ *P* value generated using a 2-tailed Student *t* test comparing mean H_E_ values across the subset of loci compared between studies.

**Table 4.** Comparison of Polyclonal Infections and MOI of Eswatini With Previously Published Studies

Country	Year	Annual Confirmed Cases per 1000^a^	Mean MOI	Polyclonal Infections, %	Reference, Publication Year
Thailand	2002–2007	0.5	…	28	Ferreira et al, 2002 [39]
Eswatini	2014–2016	1	2.2^b^ (2.7^c^)	67^b^ (74^c^)	This study
Indonesia (Papua)	2011–2014	1.5	1.2^c^	17	Pava et al, 2017 [41]
Peru	2003–2007	3	…	34	Branch et al, 2011 [42]
Ghana	2003–2004	28	2.3^d^	…	Agyeman-Budu, 2013 [43]
Mali	2012–2015	70	2.6^c^	59	Auburn et al, 2012 [15]
Burkina Faso	2012	248	3.0^c^	…	Auburn et al, 2012 [15]
Southern Mozambique (Maputo Province)	1997–1999	312	1.8^d^	49	Mayor et al, 2003 [44]

Abbreviation: MOI, multiplicity of infection.

^a^Estimates from the World Health Organization Malaria Report. Year 2000 incidence estimates are reported for studies conducted before 2000. Malaria incidence in Eswatini between 2014 and 2016 was estimated at 1 cases per 1000 person-years.

^b^Calculated from the second-highest MOI.

^c^Calculated from maximum alleles found at any 1 microsatellite.

^d^Calculated from *msp-2* typing.

### Imported Cases Are More Complex Than Local Cases

**Figure 3.**
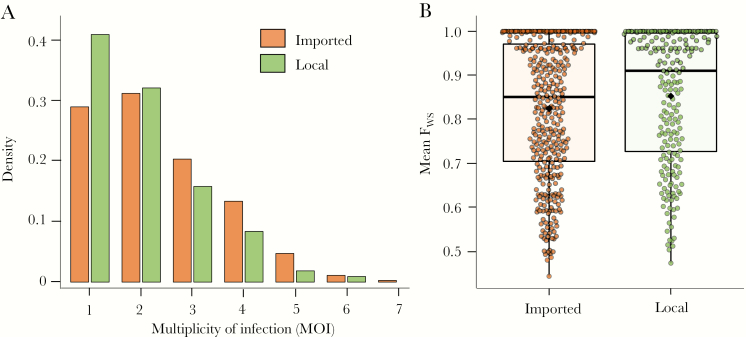
Distribution of multiplicity of infection (MOI) (*A*) and mean within-host infection fixation index (F_WS_) (*B*) by imported and locally acquired cases. Orange indicates the distribution of MOI and F_WS_ among imported cases and green indicates distribution among locally acquired cases. *B*, Black horizontal line indicates the median, diamonds the mean, and the upper and lower bounds of the box indicate the 25th and 75th quartiles.

Global measures of population-level and within-host genetic diversity were compared between imported and locally acquired cases, classifications of which were determined by travel history. Population-level diversity was similar between imported and locally acquired cases (Table 2), with little evidence of genetic differentiation (G′ _ST_ = 0.013). Despite the similarities observed in population-level genetic diversity, infections among locally acquired cases were less complex than imported cases (ie, exhibited lower within-host diversity). A larger proportion of local cases were monoclonal infections compared to imported cases (41% vs 29%; *P* = .004), and had a slightly lower mean MOI (2.0 vs 2.4; *P* = .004) (Table 1; Figure 3). Mean F_WS_ was marginally higher among local cases compared to imported cases (0.85 vs 0.82; *P* = .03). Similarly, a marginally higher proportion of local cases were considered clonal infections by this measure; 44% of local cases had a mean F_WS_ greater than 0.95 compared to 34% of imported cases (*P *= .018). Results from sensitivity analyses that estimated within-host diversity in the original case population (n = 880) showed similar patterns of within-host diversity between imported and local cases (Supplementary Table 5).

### Inclusion of MOI and F_WS_ Does Not Meaningfully Improve Discrimination Between Imported and Local Cases

Given the marginally, but statistically significant, differences in MOI and Fws between local and imported cases, we evaluated whether these metrics could predict whether a case was local versus imported, because travel history data are not always available or accurate. Compared to a base model that included district of residence, season, passively versus actively detected case, age, sex, and occupation to predict malaria importation, the model that included F_WS_ and MOI marginally improved the goodness of fit (log-likelihood *Χ*^2^ statistic difference = +8.17; *P *= .017), but had negligible effects on improving the discrimination between imported and local cases (area under the curve difference = +0.002; *P *= .6) (Supplementary Figure 3).

## Discussion

Despite sustaining low malaria transmission for nearly a decade, our study demonstrated that malaria in Eswatini is largely characterized by polyclonal, complex *P. falciparum* infections and a markedly diverse parasite population. This evidence was generated by genotyping two-thirds of all documented, symptomatic and asymptomatic *P. falciparum* infections in the country over the span of two years. The genetic diversity and large effective population size reveal a *P. falciparum* population similar to those observed among higher-transmission regions of sub-Saharan Africa [12–15, 44]. Imported and local cases had similar levels of population-level diversity and though MOI was higher and F_WS_ was lower among imported cases, the magnitude of these differences did not allow for accurate discrimination between these 2 groups. This suggests that parasite diversity in Eswatini is largely dominated by its cross-border migration from neighboring, higher-transmission countries (eg, Mozambique). However, future molecular surveillance studies of parasite diversity in Mozambique should be conducted to further confirm these findings.

These data present an important caveat to the genomic thermometer framework; namely, the assumption that as malaria reaches very low levels of transmission, *P. falciparum* populations are expected to consistently exhibit low parasite diversity [8, 11, 16–20]. Although a monotonic relationship between diversity and transmission has been observed in low-transmission settings of Southeast Asia or Latin America, where importation of malaria from higher-transmission areas is uncommon [11, 16, 17, 20, 22, 45] and/or where parasites are imported from areas with low genetic diversity [19], the genetic thermometer framework fails to accurately represent countries like Eswatini. In the low-transmission setting of Eswatini, we observed an inverse relationship between genetic diversity and transmission intensity, which is consistent with the country’s malaria epidemiological patterns. It appears that substantial human mobility from neighboring higher-transmission countries [3, 4, 7] has resulted in the frequent importation of highly diverse parasites [46, 47]. This inverse relationship may become more evident as more genotyping studies from other sub-Saharan countries with similar epidemiology become available.

In this setting, further decreases in local transmission may have actually resulted in increases in parasite diversity due to: (1) an increase in the proportion of imported infections, which tend to have higher parasite diversity, and (2) shorter local transmission. Shorter chains would be expected to increase population diversity, due to fewer related parasites circulating, and to increase within-host diversity, as decreases from imported infections likely occur with each generation of local transmission (in the absence of superinfection). As more sub-Saharan African countries with similar challenges move towards malaria elimination, this apparent paradox may become the rule rather than the exception and, as such, these metrics of diversity may not be the most appropriate methods to assess changes in transmission intensity in these settings.

Malaria importation remains a significant barrier for eliminating malaria in many countries and is further complicated by the difficulty in accurately distinguishing between imported and locally acquired infections. In Eswatini, imported and local infections had similar levels of population-level genetic diversity, with little evidence of genetic differentiation between groups. Imported cases displayed significantly higher within-host diversity compared to local cases, but these differences were minimal and likely due to the limited length of local transmission chains, as described above. As a result, these differences were too small to discriminate between imported and local infections.

One particular strength of the study was its large sample size, allowing for the most extensive and dense capture of genotyped cases from any low-transmission setting to date. Though the study was able to successfully genotype 66% of all documented cases and included sensitivity analyses which suggested that the estimated within-host diversity metrics were representative of the total case population (n = 880), our results may not be generalizable to infections not detected through the malaria surveillance system (eg, asymptomatic infections). However, the study was able to evaluate 80 cases captured through RACD, which demonstrated no differences with symptomatic cases. Second, though imported and local infections were classified using detailed travel history information and demographic factors by trained malaria surveillance officers and study team members, it is possible that cases may have been misclassified and may have biased the discriminatory capacity of MOI towards the null. Third, in contrast to comparisons of H_E_, which were recalculated and standardized between studies, comparisons of within-host genetic diversity between Eswatini and other studies may be limited in interpretability as different loci and methods were used to calculate MOI and should be interpreted as general trends without focusing on individual comparisons.

In conclusion, these findings demonstrate that *P. falciparum* genetic diversity and transmission intensity do not always follow a simple, monotonic relationship and suggest that the *P. falciparum* parasite population in an eliminating country with strong connectivity to higher-transmission countries is likely to be highly diverse. Thus, a more nuanced approach must be taken when interpreting the results from genetic analyses, particularly as a means of monitoring and evaluating the impact of malaria interventions in these settings. In countries such as Eswatini, where transmission is largely dominated by the importation of cases from higher-transmission countries, traditional metrics derived from genotyping data, such as MOI, may not be informative. In these settings, detailed characterization of *P. falciparum* transmission, or interruption thereof, may be better achieved through a more comprehensive analysis of transmission networks.

## Supplementary Data

Supplementary materials are available at *The Journal of Infectious Diseases* online. Consisting of data provided by the authors to benefit the reader, the posted materials are not copyedited and are the sole responsibility of the authors, so questions or comments should be addressed to the corresponding author.

jiz305_suppl_Supplementary_Material

## Notes

***Acknowledgments.*** The authors thank the participants for contributing their samples to the study; the health worker staff who performed sample and data collection; and the Eswatini National Malaria Program and the Clinton Health Access Initiative for their support in data collection.

***Disclaimer***. The funders of the study had no role in the study design, data collection, data interpretation, or writing of the manuscript. The authors had final responsibility for the decision to submit for publication.

***Financial support.*** This work was supported by the Bill and Melinda Gates Foundation (grant number OPP1132226); the National Institutes of Health/National Institute of Allergy and Infectious Diseases (grant number AI101012); and Burroughs Wellcome Fund/American Society of Tropical Medicine and Hygiene (grant number A120079); surveillance activities were also supported by the Eswatini Ministry of Health, partly through funding from the Global Fund to Fight AIDS, Tuberculosis, and Malaria.

***Potential conflicts of interest.*** All authors: No reported conflicts of interest. All authors have submitted the ICMJE Form for Disclosure of Potential Conflicts of Interest. Conflicts that the editors consider relevant to the content of the manuscript have been disclosed.
