# Supplementary material for: High Genetic Diversity of Plasmodium falciparum in the Low-Transmission Setting of the Kingdom of Eswatini
Source: J Infect Dis. 2019 Jul 4;220(8):1346–54. doi: 10.1093/infdis/jiz305 (PMC6743842; doi:10.1093/infdis/jiz305)

Supplementary Tables and Figures

Supplementary Table 1. Default user-fitted parameters for microSPAT software.

Parameter	Description	Default Value
**Scanning Parameters**		
Maxima Window	Final filter to remove close peaks. For each peak identified, take only largest peaks that fall within window size of each other	10
Relative Maximum Window	Number of points on each side of prospective peak to identify relative maximum	6
Smoothing Window	Window size passed to the Savitzky-Golay filter	11
Smoothing Order	Order of the polynomial used in the Savitzky-Golay filter to smooth the data	7
Tophat Factor	Size factor to apply the white-tophat filter. Tophat filter footprint size is (Tophat Factor)*(length(signal))	0.005
Filtering Parameters		
Min Peak Height	Peaks with peak height lower than Min Peak Height are filtered out	300
Max Peak Height	Peaks with peak height greater than Max Peak Height are filtered out	40000
Min Peak Height Ratio	Secondary peaks with a height ratio compared to the primary peak less than Min Peak Height Ratio are filtered out	0
Max Bleedthrough Ratio	Let signal strength be the sum of the signal from -1 to +1 indices of a peak. Bleedthrough ratio is defined as the peak signal strength divided by the maximum signal strength of other channels in the same well at the same peak index. Peaks with a bleedthrough ratio greater than Max Bleedthrough Ratio are filtered out	10
Max Crosstalk Ratio	Crosstalk ratio is defined as the peak signal strength divided by the maximum signal strength of other channels in the same color in surrounding wells (surrounding wells defined as surrounding capillaries as they feed into the machine, not surrounding wells within the plate.)	10
Min Peak Distance	Peaks that are less than Min Peak Distance away (in nucleotides) are filtered out, leaving only the tallest peaks	2.5
**Genotyping Parameters**		
Min Relative Peak Height	Peaks with a height relative to the tallest peak that do not exceed Min Relative Peak Height are classified as artifact	0.03
Min Absolute Peak Height	Minimum peak height for a peak to not be classified as artifact	500 (1000)
Bleedthrough Limit	Maximum bleedthrough ratio before a peak is classified as bleedthrough	2
Crosstalk Limit	Maximum crosstalk ratio before a peak is classified as crosstalk	2
Failure Threshold	At least one peak's height must exceed the failure threshold, otherwise the run is deemed a failure and rejected	800
Soft Artifact SD Limit	Peaks with a height that falls within the Soft Artifact SD Limit times the artifact error plus the estimated artifact contribution have a peak probability calculated when probabilistic annotation is enabled	6
Hard Artifact SD Limit	Peaks with a height minus (artifact contribution + artifact error * Hard Artifact SD Limit) < Min Absolute Peak Height are classified as artifact	0

Supplementary Tables and Figures

Genotyping Probability Threshold	Peaks with a probability less than the Genotyping Probability Threshold are classified as artifact	0.95
Iterative Probability Threshold	Peaks with a probability less than the Bootstrap Probability Threshold are culled during the iterative peak frequency and sample MOI estimation steps during probabilistic peak annotation	0.99
Off-scale Threshold	Runs with a peak that exceeds the Off-scale Threshold are flagged as off-scale and are avoided if possible	32000

* The default minimum absolute peak height was 500 florescence units, except for PfPK2, TA81, PolyA, and PFG377, where setting was raised to 1000.

Supplementary Table 2. Characteristics of genotyped and non-genotyped *Plasmodium falciparum* cases detected in Eswatini between July 2014 and June 2016.

Characteristics	All cases (n=880)	Genotyped (n=582)	Not genotyped[a] (n=298)	p-value[b]
Detection method				<0.0001
Passive surveillance	725 (82.4)	502 (86.3)	223 (74.8)	
RACD	155 (17.6)	80 (13.7)	75 (25.2)	
Season				<0.0001
2014-2015	635 (72.2)	389 (66.8)	246 (82.6)	
2015-2016	245 (27.8)	193 (33.2)	52 (17.4)	
District				<0.0001
Hhohho	157 (19.8)	115 (22.4)	42 (14.5)	
Lubombo	389 (48.5)	235 (45.8)	154 (53.3)	
Manzini	239 (29.8)	158 (30.8)	81 (28.0)	
Shiselweni	17 (2.1)	5 (1.0)	12 (4.2)	
Case classification				<0.0001
Imported	485 (55.1)	359 (61.7)	126 (42.3)	
Locally acquired	376 (42.7)	215 (36.9)	161 (54.0)	
Unknown	19 (2.2)	8 (1.4)	11 (3.7)	
Gender				0.0088
Female	222 (27.2)	128 (24.2)	94 (33.0)	
Male	593 (72.8)	402 (75.8)	191 (67.0)	
Age, median (IQR)	25.3 (11.3-37.1)	26.6 (11.9-37.1)	23.9 (9.8-37.2)	0.31
Occupation				0.0003
Child	135 (15.6)	75 (13.1)	60 (20.5)	
Farming/Agriculture	93 (10.8)	64 (11.2)	29 (9.9)	
Manual Laborer	103 (11.8)	77 (13.5)	25 (8.6)	
Manufacturing/Factory	61 (7.1)	52 (9.1)	9 (3.1)	
Small-market trader	66 (7.6)	50 (8.7)	16 (5.5)	
Student	186 (21.5)	127 (22.0)	59 (20.2)	
Unemployed	170 (19.7)	97 (17.0)	73 (25.0)	
Other	48 (5.9)	30 (5.2)	21 (7.2)	

RACD = reactive case detection; IQR = interquartile range

Note: Values may not add up to total N due to missing values. Values for categorical variables represent N (Column %) and continuous variables show median (IQR).

[a] Non-genotyped samples included genotype failures (25%), samples where dried blood samples were either not collected or not available for DNA extraction (72%), and samples that were negative by quantitative PCR (3%).

[b] p-value generated using Chi-squared test for categorical variables or Kruskal-Wallis test for continuous variables.

Supplementary Figure 1. Density plot of propensity scores (e.g. estimated probabilities of being genotyped) stratified by actually observed genotyped and non-genotyped cases.

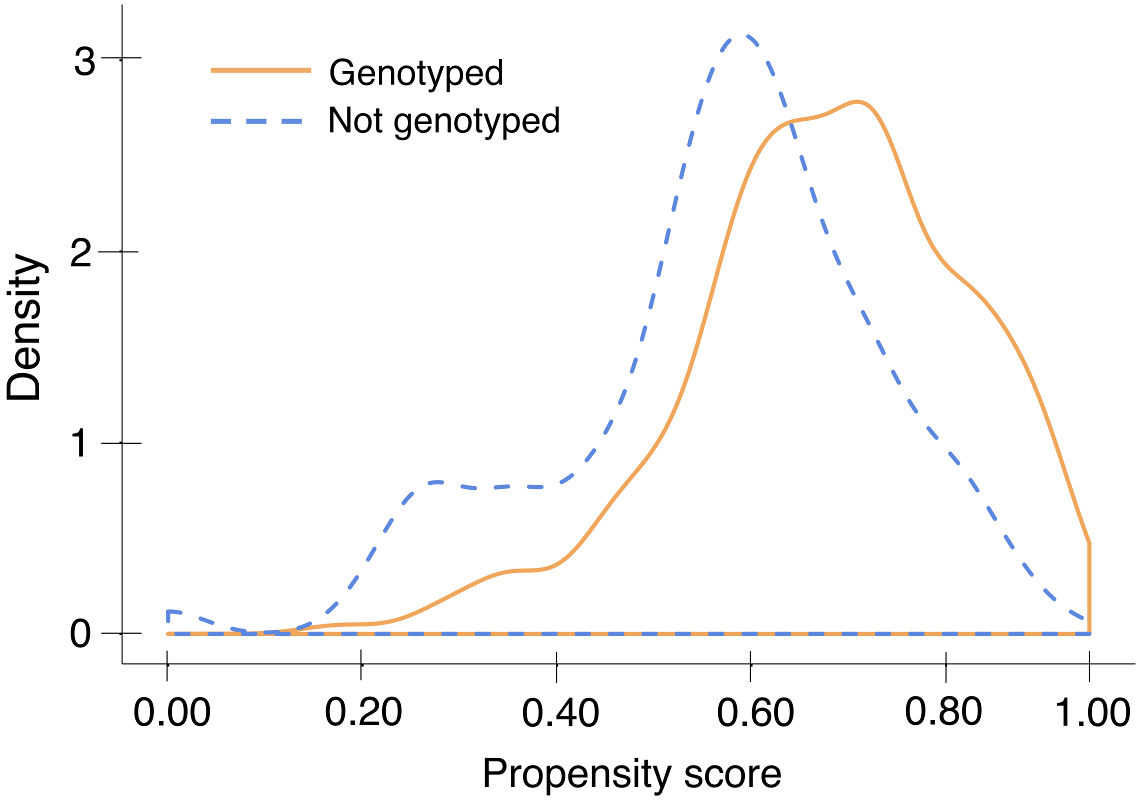

Supplementary Figure 2. Distribution of mean F_{WS} by MOI among genotyped Eswatini samples.

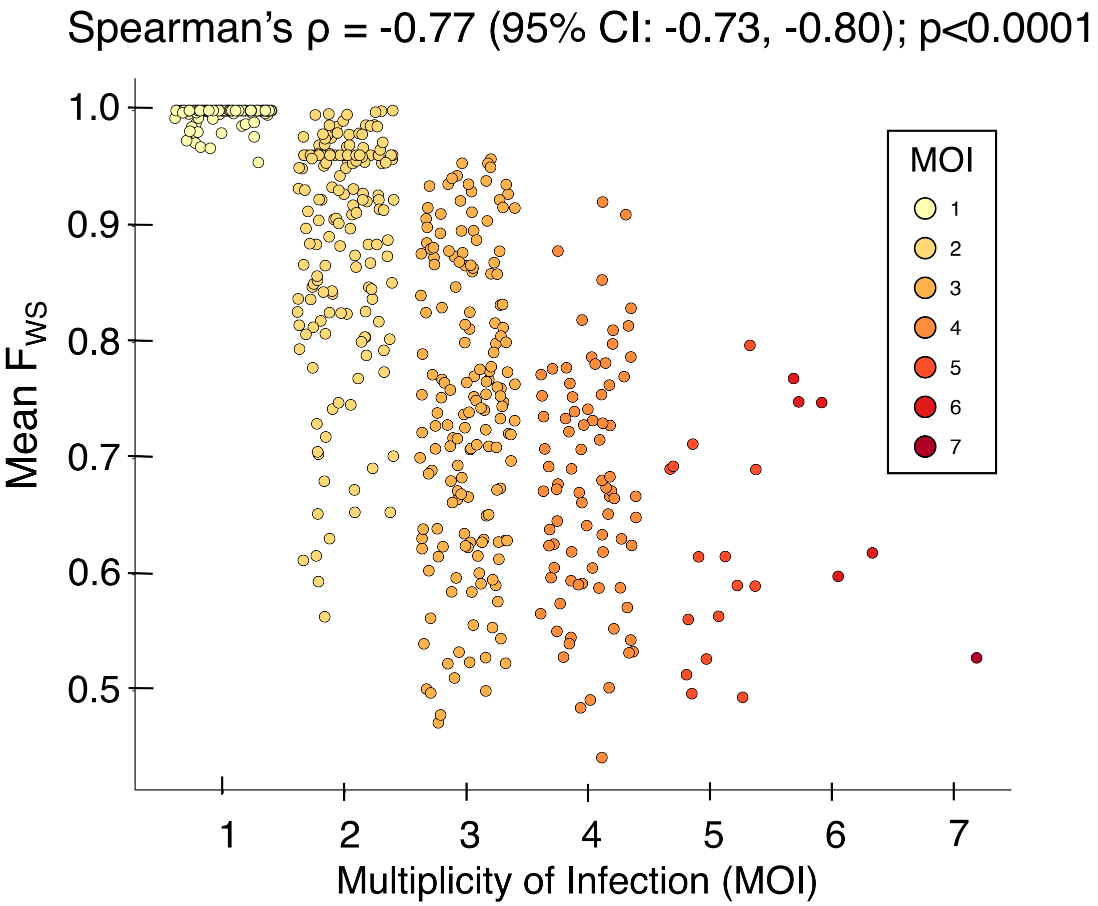

Supplementary Table 3. Expected Heterozygosity (H_E) Assessed at 26 Microsatellites.

Locus	Total	Case classification	
		Imported	Local
Ara2	0.862	0.864	0.848
AS1	0.546	0.533	0.558
AS2	0.770	0.787	0.743
AS3	0.858	0.861	0.858
AS7	0.533	0.511	0.600
AS8	0.572	0.547	0.605
AS11	0.715	0.711	0.726
AS12	0.528	0.511	0.544
AS14	0.773	0.779	0.764
AS15	0.848	0.839	0.854
AS19	0.668	0.663	0.675
AS21	0.520	0.529	0.492
AS25	0.864	0.859	0.864
AS31	0.860	0.860	0.860
AS32	0.718	0.686	0.722
AS34	0.551	0.563	0.526
B7M19	0.604	0.605	0.605
PFG377	0.655	0.678	0.606
PfPK2	0.919	0.924	0.910
PolyA	0.931	0.935	0.905
TA1	0.897	0.894	0.883
TA40	0.898	0.896	0.910
TA60	0.827	0.830	0.818
TA81	0.857	0.853	0.864
TA87	0.883	0.883	0.877
TA109	0.818	0.820	0.824
**Mean H_E ± SD**	0.75 ± 0.14	0.75 ± 0.15	0.75 ± 0.14

Supplementary Table 4. The Number of Unique Alleles (A) Assessed at 26 Microsatellite Loci.

Locus	Total	Case Classification	
		Imported	Local
Ara2	13	13	13
AS1	7	7	6
AS2	11	11	11
AS3	11	11	11
AS7	13	11	13
AS8	7	5	6
AS11	14	14	10
AS12	8	6	6
AS14	15	14	15
AS15	14	14	13
AS19	17	16	15
AS21	9	7	5
AS25	23	21	20
AS31	24	24	19
AS32	21	18	16
AS34	8	7	4
B7M19	7	7	5
PFG377	7	7	7
PfPK2	22	21	22
PolyA	32	30	25
TA1	29	29	23
TA40	24	22	23
TA60	12	11	10
TA81	16	16	14
TA87	17	16	15
TA109	20	18	15
**Mean A ± SD**	15.4 ± 7.1	14.5 ± 7.0	13.2 ± 6.2

Supplementary Table 5. Propensity-score weighted MOI and mean F_{WS} from sensitivity analyses

Measures of within-host diversity	Total	Case classification		
		Imported	Local	p-value[a]
% polyclonal infections (95% CI)	75 (72-79)	78 (71-80)	70 (63-76)	0.032
Mean MOI ± SD	2.4 ± 1.2	2.5 ± 1.2	2.3 ± 1.1	0.19
Mean F_{WS} ± SD	0.838 ± 0.154	0.83 ± 0.24	0.86 ± 0.20	0.036

[a] p-values calculated from simple linear regression with probability weights.

Supplementary Figure 3. Receiver operating characteristic (ROC) curves of case classification models. Blue line indicates ROC curve of model with MOI and F_{WS} as covariates. Orange line indicates ROC curve of model with only epidemiological (epi) covariates. Green line indicates ROC curve of full model with both epi and genetic covariates. The null hypothesis (black dashed line) is that the area under the curve (AUC) equals 0.5.

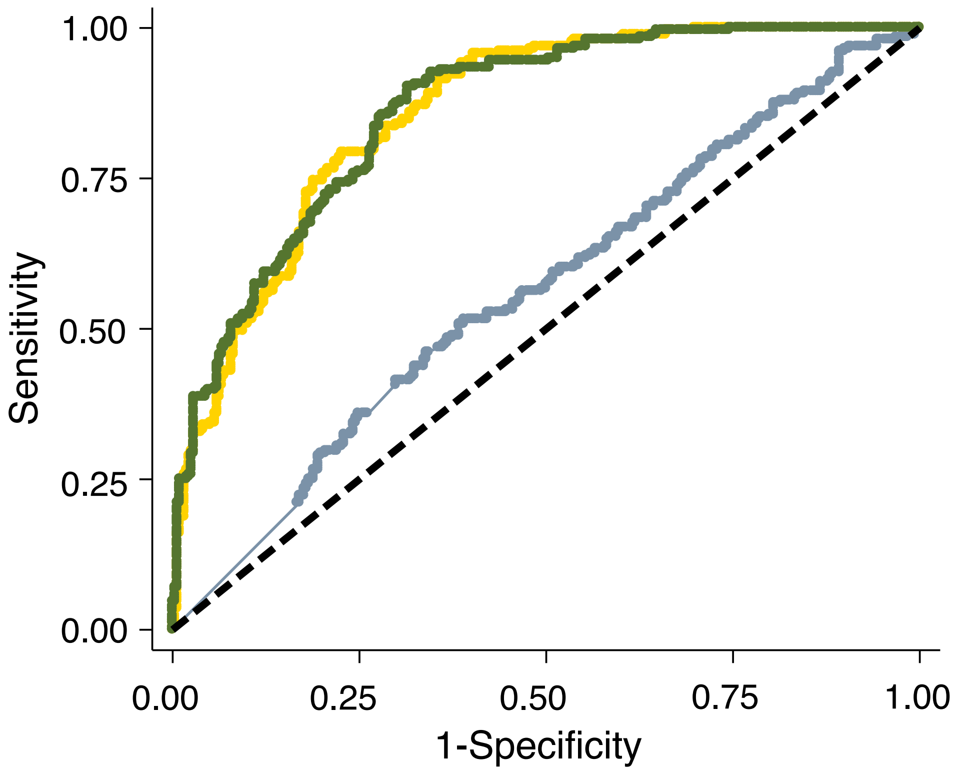

Supplement: jiz305_suppl_Supplementary_Material [file jiz305_suppl_supplementary_material.pdf]
